# Evaluation of an Accelerometer-Based Device for Testing the Softness of Bedding Materials Used for Livestock

**DOI:** 10.3390/s22228912

**Published:** 2022-11-18

**Authors:** Karina Regina Weimar, Barbara Pichlbauer, Christian Guse, Johannes Peter Schramel, Christian Peham, Marc Drillich, Michael Iwersen

**Affiliations:** 1 Clinical Unit for Herd Health Management in Ruminants, University Clinic for Ruminants, Department for Farm Animals and Veterinary Public Health, University of Veterinary Medicine Vienna, 1210 Vienna, Austria; 2 Movement Science Group, University Equine Hospital, Department for Small Animals and Horses, University of Veterinary Medicine Vienna, 1210 Vienna, Austria

**Keywords:** cows, bedding materials, surface testing, sensor system, accelerometer, evaluation, Vienna Surface Tester

## Abstract

Lying is a high priority behavior for dairy cows. As the quality of cubicles can influence their lying time, the interest in finding objective methods to assess the quality of floors has increased substantially over recent decades. This study aimed to evaluate a technical device for measuring elastic properties of floors for the application to bedding materials for cows. Ten different floor types were used: horse manure, recycled manure solids, bark mulch, sand, sawdust, and three different rubber mats. Horse manure and bark mulch were additionally tested with chopped straw as a top layer. Two devices of the same kind and two examiners were available for performing comparative measurements. Regression analyses and an ANOVA were conducted to compare the devices, examiners, and different surfaces. Most of the floors differed significantly from each other. Sawdust was the softest material, followed by sand and recycled manure solids. The agreement between the devices (Lin’s concordance correlation coefficient (CCC) > 0.99, Spearman’s rank correlation coefficient (r_S_) = 0.99) and examiners (CCC = 0.99, r_S_ = 0.99) was almost perfect. These findings indicate that this device can be used as a new method for assessing the softness of bedding materials for dairy cows objectively.

## 1. Introduction

The implementation of sensor technologies in agricultural settings is rapidly increasing, not only for the application in the field of agricultural engineering, but also in the context of livestock farming. Using sensor systems for the continuous and real-time monitoring of farm animals in order to improve management, animal health and welfare, among others, is nowadays referred to as Precision Livestock Farming (PLF) [1]. Many accelerometer-based systems aim to classify the behavior of animals in order to gain insights into health and welfare [2]. Animal behavior can be linked to environmental parameters. Bedding materials have an influence on lying times of cows, as well as on health and production parameters. Dairy cows spend approximately 8–16 h per day in the lying position [3]. This fact shows that lying behavior has a high priority for dairy cows [4,5]. Previous research found that dairy cows in tie-stalls prefer and spend more time lying in well bedded, soft, and dry cubicles [6]. Tucker and Weary [7], for example, detected that for each additional kilogram of sawdust and straw on a mattress, Holstein cows increased their daily lying time by 12 min. In a similar study, Tucker et al. [8] found that Holstein cows spend more time lying in deep-bedded sawdust and deep-bedded sand stalls than in stalls with mattresses covered with 2–3 cm of sawdust.

Numerous research studies support the various health benefits of sufficient length of lying periods and the importance of suitable bedding material. During lying, claws and joints are relieved, and claws can dry. Increased lying times are associated with improved claw health [9] and higher milk yield [10]. A previous study by Fulwider et al. [11] reported less frequent hock lesions in cows housed on deep-bedded sand compared with cows on mattresses. These findings are supported by van Gastelen et al. [12], who found that the severity of injured hocks of lactating Holstein cows was lower on farms using bedding materials such as compost, sand, and horse manure compared with farms using foam mattresses. Considering these facts, it becomes clear that the quality and management of cubicles are essential aspects in dairy farming, and farmers are responsible for taking care of their cows’ welfare, which includes comfort during resting [13].

Different methods and systems have been described to assess the quality of a lying surface. They can be distinguished by approaches that directly measure the surface properties and indirect assessments that analyze the cows’ lying behavior. Among the approaches for assessing the properties of lying surfaces directly, the “knee drop test” has to be mentioned first. This method was described by Nordlund and Cook [14] and is widely used in practice but rather subjective. In previous studies, researchers used the penetration depth of different kinds of spheres to characterize bedding materials, e.g., the mixing bowl method [6]. Wechsler et al. [15] showed that the observation of standing and lying behavior is an indirect approach to assess bedding quality that can be carried out directly in the barn or by using video recordings. In recent years, various sensor systems have been developed in order to assess the quality of lying surfaces. For example, accelerometers which can be mounted on different positions of a cow (e.g., ear, neck, fetlock) are used to draw indirect conclusions on the comfort around resting by measuring lying times of cows [13,16]. It should be mentioned in this context that lying times are individual for each animal and differ according to breed, lactation stage, and season [17]. These factors have to be taken into account when assessing the sensor-based recording of lying times.

Fulwider and Palmer [18] evaluated the Clegg Impact Soil Tester (CIST, model 95051, Lafayette Instruments, Lafayette, IN, USA) for measuring the compressibility of mattresses and rubber mats in free-stall barns, and Villettaz Robichaud et al. [19] conducted similar research for mattresses and rubber mats with and without additional bedding in tie-stall barns. The main finding of the first study was that the objective output of the device correlated with cow preferences for lying surfaces and can therefore be used for predicting cow preferences for stall bases. The latter study reported significant differences between rubber mats and rubber mats with different depths of bedding material on top.

In the present study, we used the Vienna Surface Tester (VST) to provide objective measurements of surfaces and floors as used in cattle farming. It was developed by Schramel and Peham at the University of Veterinary Medicine Vienna, Austria. So far, the device has primarily been used for measuring the mechanical properties of sport surfaces such as turf or riding arenas, but previous work has already tested different bedding materials used for horses, such as straw and sawdust [20].

The main objective of this study was to evaluate the device for measuring the softness (represented by the stiffness or spring rate, [kN∙m^−1^]) of different bedding materials used on cattle farms. Another aim was to investigate the agreement between two devices and two trained examiners. The main conclusion of this work is that the VST can be used to measure the stiffness of different bedding materials for cattle. Therefore, it can be seen as a new technology that enables the objective comparison of lying surfaces in livestock husbandry.

## 2. Materials and Methods

### 2.1. Experimental Sites

The study was conducted on two farms. The first part took place in July 2021 on a commercial dairy farm in northern Germany, using the following bedding materials: horse manure (HM), horse manure with chopped straw (HMS), recycled manure solids (REC), bark mulch (BM), bark mulch with chopped straw (BMS), and a soft rubber mat (SRM I). The second part of the study took place at the Teaching and Research Farm (VetFarm) of the University of Veterinary Medicine Vienna in Austria in February 2022. The following surfaces were tested there: sand (SA), a second soft rubber mat (SRM II), a hard rubber mat (HRM), and sawdust (SAW). No animals were involved in this study.

### 2.2. Vienna Surface Tester

**Figure 1:**
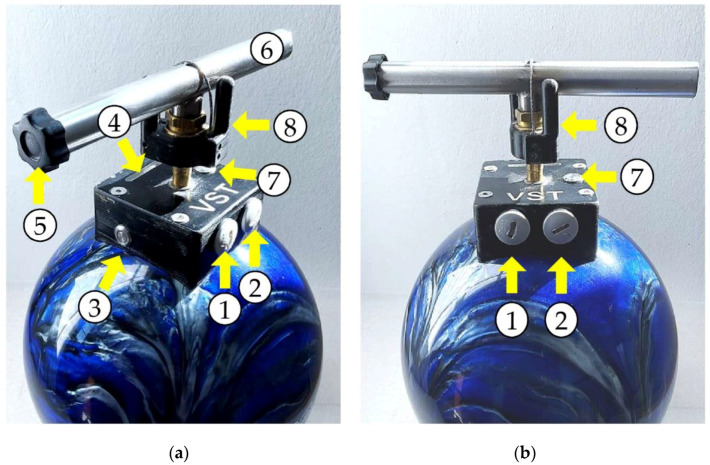
The figure shows the Vienna Surface Tester (VST) and its controls from the side view (**a**) and the front view (**b**): Two blind plugs on the side cover the micro-SD card slide (1) and the micro-USB connector (2) for charging the device. The power button is located on the side (3). For each drop, the examiner has to hold the ball with the handle (6) still above the ground and press the arm button (7) with the middle finger. The color LED bar (4) turns blue, which indicates that the device is ready. Then the examiner has to turn the release mechanism (8) clockwise within four seconds to perform the measurement. The safety screw (5) has to be fastened on top of the handle after measuring to prevent unintentional dropping.

Figure 1 shows the Vienna Surface Tester (VST) with its different elements for operating. It is a sphere with a weight of 6.15 kg and a diameter of 20.4 cm. The device is equipped with two accelerometers and calculates impact velocity, impact acceleration (Gmax), stiffness (spring rate), penetration depth, resonance frequency, and the E-modulus. The sampling rate of the accelerometers is 38 kHz. More technical details can be found in the patent specification [21]. In this study, the parameters “stiffness” and “impact velocity” were of major interest for analysis. The two devices used in this study were the wireless second generation (Vienna Surface Tester Mark2, JP Schramel, Vetmeduni Vienna, V1.0 March 2020).

According to the standard operating procedure provided by the manufacturer, the sphere has to be dropped from random heights between 5–85 cm above ground to cover a range of impact velocities between approximately 1 m∙s^−1^ and 4 m∙s^−1^. At least 14 drops of the device are necessary to complete one measurement. This is required to determine the surface parameters under different loads and to take the variability of natural surfaces into account. As the impact of the sphere can alter the floor compaction, each drop has to be placed at another spot of one measurement plot (e.g., area of one square meter). A color light-emitting diode (LED) bar indicates the status of measured data. It displays whether two valid drops were performed successfully at all required height-levels. Data are stored on a micro-SD card in csv format. The rechargeable lithium-polymer battery lasts for approximately six hours of continuous use. The first drop should be carried out at a height of approximately 20 cm above the ground. Subsequently, the examiner starts at the lowest level and increases the height stepwise after two successful drops indicated by the LED lamp.

### 2.3. Study Design

**Figure 2:**
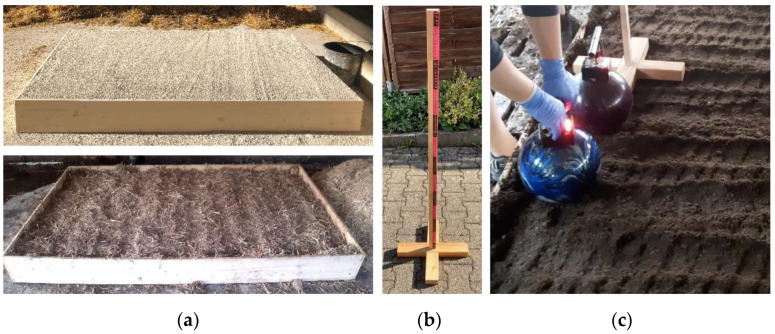
This figure shows the test cubicle filled with sand in the upper picture and horse manure in the lower picture (**a**) as well as the measuring tool for estimating the height for dropping the device (**b**). (**c**) presents the measurement procedure: One of the examiners performs a measurement on one bedding material (here: recycled manure solids) using both devices alternatingly.

For the entire experiment, ten different types of floors, two devices, and two examiners were available. For the standardization of the experiment, a wooden frame with the dimensions of a standard cubicle (20 × 200 × 120 cm) was used to surround the different materials (Figure 2a) and ensure a standardized amount and thickness of each material. A wooden slat of 124 cm length was used as a measuring tool (Figure 2b) to facilitate dropping the device from seven distinct heights. The appropriate height-levels for the drops were determined in preliminary tests and were at 40, 50, 60, 70, 90, 100, and 120 cm. On REC, it had to be dropped from 35 instead of 40 cm and 45 instead of 50 cm to activate the first and second LED lamp, respectively.

**Figure 3:**
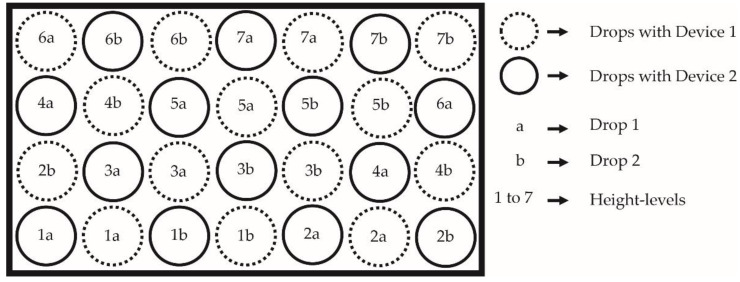
The figure illustrates the test cubicle and the standardized sequence of drops per measurement and examiner with both devices. The rectangle represents the test cubicle and the circles are the spots where the devices were dropped.

Each examiner carried out three comparative measurements with both devices per floor, resulting in 12 single measurements per floor in total. Figure 3 shows the scheme of the procedure of measuring the same floor with both devices. One comparative measurement performed by one examiner required dropping both devices alternatingly across the test cubicle, starting at low levels and increasing the height stepwise, as it is shown in Figure 2c. This approach was chosen to enable direct comparability of the devices, as the surface of most of the materials visibly changed after each drop.

### 2.4. Material Characteristics and Preparations

**Figure 4:**
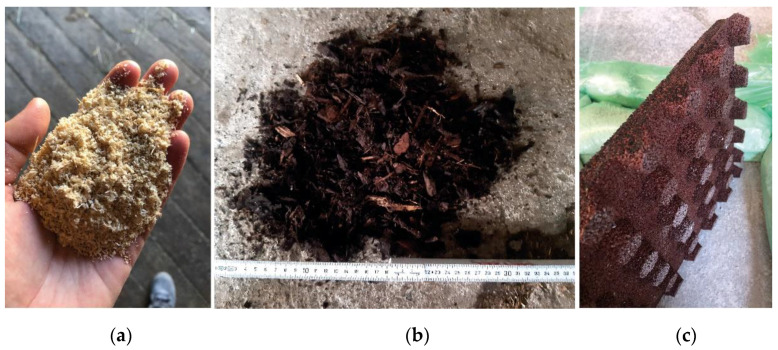
Three tested materials: sawdust (SAW) (**a**), bark mulch (BM) (**b**), and the soft rubber mat II (SRM II) (**c**). The rubber mat is presented from the lower side to display the rubber feet.

In this study, dry angular quartz sand with a particle diameter up to 4 mm was used. SAW had a particle size of a few millimeters and a homogeneous structure, as shown in Figure 4a. These two materials were prepared as “loose filling”: They were manually shoveled into the wooden frame until it was completely filled (Figure 2a, upper picture), without compacting the material. Between the single measurements on SAW, a pitchfork was used to loosen the material in the entire test cubicle and return it to the initial position. This was not possible with SA in the same way. For loosening the surface of SA between the measurements, the tines of a pitchfork were put approximately 10 cm deep into the sand and moved horizontally through this top layer. This procedure was performed four times across the entire test cubicle, resulting in four different directions of movement. This required significantly more force and did not restore the initial setting, but it was suitable for loosening the surface.

The HM was a composite material according to the standard mixture of the commercial farm: Horse manure and lime in approximately the same weight proportions (3000–4000 kg) and 200 L of water were mixed in a feed mixing truck. The HM material produced in this manner had an inhomogeneous structure due to the horse droppings of different sizes. For the bedding material HMS, chopped straw of about 3 cm lengths was spread on top of the HM material in a layer 5 cm thick. This was done after the measurements on HM were finished. The additional straw was moistened with water, using the following guide: after compressing in the hand, the palms should be damp, but water should not escape from the straw during compressing. The BM (Figure 4b) was a commercially available brown bark mulch with an inhomogeneous particle size. Some particles were only a few millimeters, but others were approximately 12 cm by 3 cm in size. For the material BMS, chopped straw of about 12 cm lengths was used to prepare a top layer according to the procedure in HMS. The straw layer had a thickness of 5 cm and was added on top of the BM after all measurements on the BM were finished.

The REC was produced at the on-farm biogas plant. It was one of the standard bedding materials on the commercial farm. The REC had a particle size of a few millimeters and a homogeneous structure, similar to SAW. HM, HMS, BM, BMS, and REC were prepared using the procedure of “filling and compaction”: The material was shoveled into the test cubicle in several layers. Each layer was compacted before adding the next layer until it reached the rim. Between the single measurements, the top 5 cm were loosened with a rake.

The SRM I (Regupol B10, Regupol BSW GmbH, Bad Berleburg, Germany) had a size of 25 × 25 × 5 cm. The SRM II (Regupol BSW GmbH) had a size of 25 × 25 × 2 cm and additional rubber feet (measuring 2 × 2 × 2 cm) distributed evenly across the entire bottom (Figure 4c). Both soft rubber mats consisted of a rubber granulate bonded with polyurethane. The HRM was the model Cura P (Kraiburg rubber company, Tittmoning, Germany). This type of rubber mat is used as the floor material in the barn alleys on VetFarm. The rubber mats did not need specific preparations before and between measurements. SRM I and SRM II were placed one after another on an even concrete floor, surrounded by padding material.

The two materials SA and SAW could not initially be measured with the default software setting of the VST in the first part of the experiment in northern Germany. This was due to a certain threshold (30 kN∙m^−1^) that is integrated into the software to automatically exclude erroneous readings. By default, values that are below this threshold are not stored. Hence, for the second part of the study, the manufacturer adjusted the threshold in order to enable measurements on SA and SAW. The SRM II and HRM were also measured with the adjusted setting, as they were also tested in the second part of the experiment at VetFarm.

### 2.5. Data Pre-Processing

Data from both VST devices were merged in Microsoft Excel (MS Excel 2016, Microsoft Corporation, Redmond, DC, USA), according to the chronology of the experimental procedure. Coding variables were added to identify the device, the examiner, the floor type, the drops belonging to one measurement (continuous number), and the measurements from each device for direct comparison (measurement number). An index was assigned to each single drop according to the chronology of the drops. The above-mentioned documentation of the LED lamp and the height of the measurement aid were added manually for each drop. Comments regarding issues with dropping the device or technical difficulties were documented in the spreadsheet. In a next step, a validity check of the data was carried out. Drops were excluded if the notes indicated that there had been issues during measurements and if any data were missing. For the direct comparison of the two devices, the drops were arranged in pairs, sorted by “measurement number” and impact velocity. Drops were also excluded if more than two drops per height-level (indicated by the LED number) existed. In this case, the chronologically last one was always excluded. At the end, each drop of “Device 1” had a paired drop of “Device 2” at the same height-level (indicated by the LED number).

### 2.6. Statistical Analysis

Statistical analysis was carried out with SPSS (version 27, IBM Corporation, Armonk, NY, USA) and with R (version 4.0.4, Copyright 2021 The R Foundation for Statistical Computing) software.

Data were analyzed in three levels. The first level was the results from the single drops per measurement, to which we will refer as “measured stiffness values” (mS) from now on. Each full measurement had 14 drops at maximum, in some cases less due to the above-mentioned criteria for valid data.

The second level was the measurement level, for which the mS (11–14 per measurement) served to calculate three stiffness values for each measurement at standardized height levels, hereinafter referred to as “calculated stiffness values” (cS). In detail, the single drops of each measurement were used to conduct a regression analysis, setting the “stiffness” (S) as the dependent variable and the “impact velocity” (V) as the independent variable. The three impact velocity values for the following calculations were set as V = 2 m∙s^−1^ (V2), V = 3 m∙s^−1^ (V3), and V = 4 m∙s^−1^ (V4). Subsequently, the regression coefficients (slope and intercept) and the three predefined V-values were entered into the formula of the linear equation:

cS = a × V + b,(1)

where a is the slope and b is the intercept of the linear equation.

The three cS per measurement were considered as the output for each measurement.

The third level was the floor level, for which the above-mentioned procedure with regression analysis was carried out, not for each measurement, but for each floor type separately. This approach was chosen to standardize the comparison of measurements and floor types and to take into account the height from which the device was dropped when assessing the stiffness.

### 2.7. Comparison of Devices and Examiners

The mS of the single drops of both devices were tested for normal distribution using the Shapiro–Wilk test. In the first step, the agreement of both devices was calculated by computing linear regression analysis, Lin’s concordance correlation coefficient (CCC), and Spearman’s rank correlation coefficient (r_S_) for the paired drops of Device 1 and Device 2 across the entire dataset. In addition, a Bland–Altman plot was used for a graphical analysis of agreement. The bias was computed as the mean of the differences between the single drops, the limits of agreement were calculated as the bias ± 1.96 x standard deviation of the differences. 

For the comparison of the two examiners with each other, measurements on rubber mats (SRM I, SRM II, and HRM) with the same device were selected for analysis to ensure constant floor conditions. The study design for the comparison of the two devices on other materials did not allow the direct comparison between examiners, as the surface had to be restored and prepared between the measurements by different examiners.

### 2.8. Variation between Measurements and Comparison of Floor Types

To assess the repeatability of the results from the sensor system, cS from measurements on rubber mats (SRM I, SRM II, and HRM) were used. Considering the results from the comparison of devices and examiners, the measurements of both devices and both examiners could be analyzed together in this step. Consequently, 12 cS were available per rubber mat for each of the predefined V-values (108 in total). Mean and standard deviation as well as minimum and maximum of these 12 cS were calculated. Additionally, the coefficient of variation was computed for each floor type and V separately. 

For the comparison of the different floor types, cS per floor of both devices and both examiners were also analyzed together. Data from all different floor types were included to this analysis. The Shapiro–Wilk test was used to test the cS data for normal distribution at floor-level. An ANOVA was conducted using the Bonferroni correction as a post hoc test. The significance level was set at 0.05.

## 3. Results

After data pre-processing, different numbers of data points were available for the different research questions. For the comparison between the two devices, 795 paired mS were used in total and for the comparison between the two examiners, and 250 paired mS were available. According to the Shapiro–Wilk test, the mS were not normally distributed.

The 12 cS per rubber mat type and V-value were used to calculate the variation between measurements within each group of V-values. At floor-level, 12 valid cS (three measurements by two examiners using two devices) were available per floor type for each V-value. For BMS, just eight cS could be included in the analysis due to technical issues (defect of release mechanism). This resulted in a total of 348 cS for the comparison of the different bedding materials, which were grouped by V-values.

### 3.1. Comparison of Devices and Examiners

**Figure 5:**
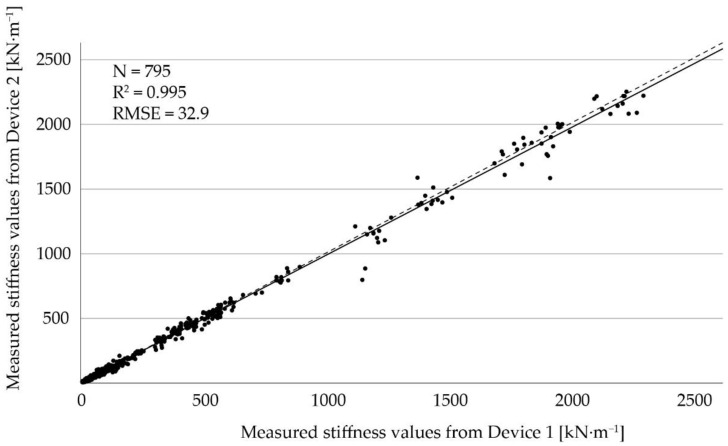
Linear regression of stiffness values from Device 1 compared to Device 2. The solid line indicates the regression line and the dashed line represents the angle bisector.

A high correlation of mS between the two devices with CCC > 0.99 and r_S_ = 0.99 (n = 795, for both) was observed. The linear regression line observed for the two devices is presented in Figure 5. The coefficient of determination was R^2^ = 0.995 and the root mean square error (RMSE) was 32.9 kN∙m^−1^, which indicates a strong agreement between the two devices.

**Figure 6:**
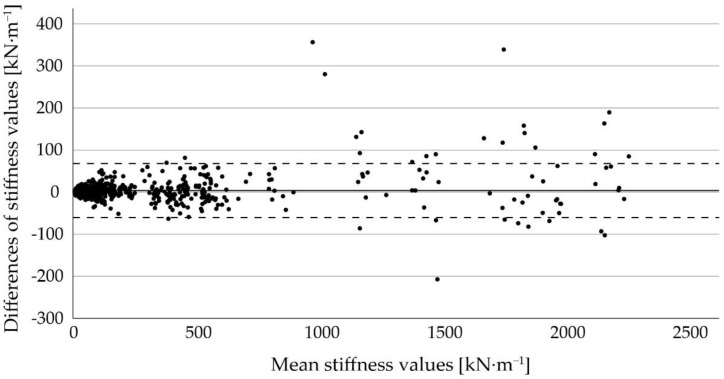
Bland–Altman plot for comparison of the stiffness values [kN∙m^−1^] of the two devices. The solid line represents the mean difference and the dashed lines indicate the upper and lower limits of agreement.

The Bland–Altman plot in Figure 6 shows the differences of mS between the two devices against their mean across the different floor types. The mean difference was 3.8 kN∙m^−1^, with upper and lower limits of agreement of 67.9 kN∙m^−1^ and −60.3 kN∙m^−1^, respectively. Although some values can be found outside the dashed lines, especially with greater stiffness values, the majority of differences are within the limits of agreement.

**Figure 7:**
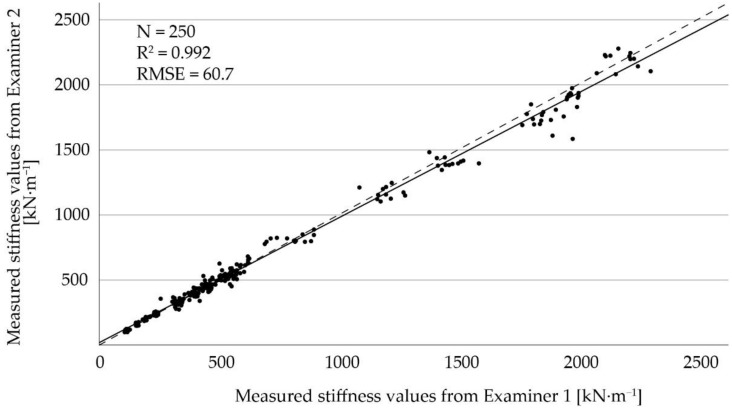
Linear regression of stiffness values from Examiner 1 compared to Examiner 2. The solid line indicates the regression line and the dashed line represents the angle bisector.

For the comparison between the two examiners, the linear regression line is shown in Figure 7. The coefficient of determination was R^2^ = 0.992 and the RMSE was 60.7 kN∙m^−1^, indicating a strong agreement between the two examiners. The correlation of mS between the two examiners was high with CCC = 0.99 and r_S_ = 0.99 (n = 250, for both).

### 3.2. Variation between Measurements and Comparison of Floor Types

For assessment of the variation between measurements, the 108 cS of the single measurements were split by V and floor type (i.e., different rubber mats), resulting in 12 measurements per group for comparison. The results are presented in Table 1. The mean and standard deviation (SD) increase with greater V-values for all the given floors, except for HRM, where the SD remains constant between V2 and V3. The ranges of the 12 cS in each group tend to be greater in harder surfaces. The coefficient of variation ranges between 0.02 and 0.05.

Table 2 provides the cS for the three different V-values (V2, V3, V4) for all bedding materials. The cS for the different floors range from 9.7–905.7 kN∙m^−1^ at V2, from 12.6–1524.9 kN∙m^−1^ at V3, and from 15.4–2144.1 kN∙m^−1^ at V4. The softest floor was SAW as opposed to HRM, which had the greatest stiffness values. SA had the lowest coefficient of determination (R^2^ = 0.1), whereas HRM had the greatest (R^2^ > 0.9). The increase of cS at greater V-values depended on the floor type, with harder floors tending to have greater differences between the three categories. Moreover, a tendency of harder bedding materials to have greater coefficients of determination can be observed, apart from SAW.

**Figure 8:**
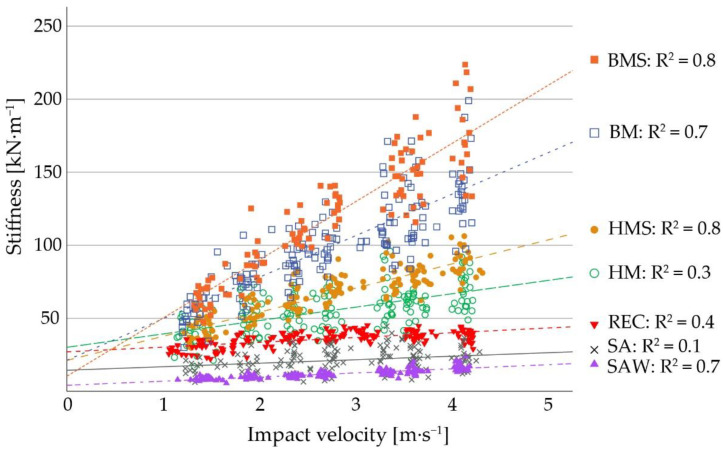
Measured stiffness values, their respective regression lines, and coefficients of determination of different bedding materials. The materials shown in this graph are bark mulch with chopped straw (BMS), bark mulch (BM), horse manure with chopped straw (HMS), horse manure (HM), recycled manure solids (REC), sand (SA), and sawdust (SAW).

Figure 8 shows the regression lines and coefficients of determination for all bedding materials that were filled into the test cubicle. According to this graph, the surface types can be assigned to three “stiffness” categories: SAW, SA, and REC form the smallest category, HM and HMS are in the middle, and BM and BMS comprise, with some distance to the middle, the group with greatest stiffness values.

**Figure 9:**
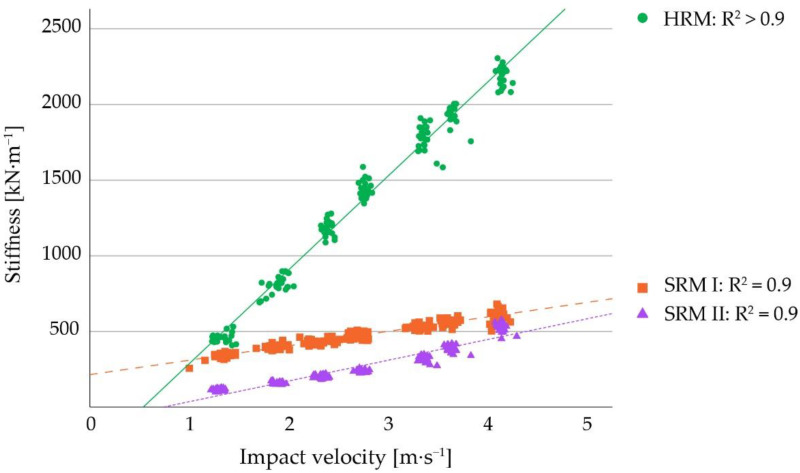
Measured stiffness values, their respective regression lines, and coefficients of determination of different rubber mats. The rubber mats shown in this graph are the first soft rubber mat (SRM I), the second soft rubber mat (SRM II), and the hard rubber mat (HRM). Note, that the stiffness scale here is 100 times greater than in Figure 8.

Figure 9 illustrates the same parameters for the three types of rubber mats in this study. When comparing the rubber mats with the other materials, HRM is more than ten times harder than BMS. This relation can be recognized also between SRM I and HM as well as between SRM II and REC, approximately.

An ANOVA was conducted for the comparison of the different bedding materials for each V separately. There was no significant difference between the stiffness values obtained for SAW and SA (V2, V3, and V4: *p* > 0.99) as well as for SA and REC (V2 and V4: *p* > 0.99; V3: *p* = 0.89) at any V. Between SAW and REC, we observed a difference at V2 and V3 (*p* = 0.01) but not at V4 (*p* = 0.16). REC and HM (V2: *p* = 0.63; V3: *p* = 0.05; V4: *p* = 0.07) as well as HM and HMS (V2 and V3: *p* > 0.99; V4: *p* = 0.69) did not differ at any V. Comparing BM and BMS, we observed no significant difference at V2 (*p* > 0.99) and V3 (*p* = 0.06), but at V4 (*p* = 0.01) we did. The other materials differed between each other at all V-values (*p* < 0.01).

## 4. Discussion

### 4.1. Comparison of Devices and Examiners

The two devices were from the same manufacturer, but the second device was a newer generation with minor adaptions (e.g., additional seals for better water protection). The almost perfect correlation coefficients as well as the results from the regression analysis indicate a strong agreement between the measurements of the two devices.

The overall mean difference obtained by Bland–Altman analysis indicates a slight overestimation of stiffness with Device 2 compared to Device 1. Some difference-values lie outside of the limits of agreement, especially at greater mean stiffness values, which were obtained from measurements on rubber mats. Based on the Bland–Altman plot, the difference between the devices increases with increasing absolute stiffness values, but there is no substantial disagreement in terms of relative differences. This is supported by the small coefficients of variation across all types of rubber mats (ranging from 0.02–0.05).

The results from the comparison between the two examiners were obtained only from measurements on rubber mats, as they do not change substantially over time [17]. The results from the regression analysis (Figure 5) and the correlation coefficients indicate a strong agreement between the examiners. Therefore, we conclude that data can be merged for analysis if generated by different trained persons. In summary, these results show no considerable disagreements in direct comparison, which is crucial concerning repeatability of measurements and comparability of data.

### 4.2. Variation between Measurements and Comparison of Floor Types

As presented in Table 1, the greatest coefficient of variation of cS from rubber mats was 0.05. Because a coefficient of variation of less than 10% is considered as excellent [22], this indicates a good repeatability and precision of the device for measuring the stiffness of surfaces.

The different bedding materials can be distinguished by the numerically different cS at given V-values per floor. Some of the floor types, however, did not have significantly different cS when compared by ANOVA. These similarities between some floors can be explained for HM and HMS as well as BM and BMS, as their physical structures were closely comparable to each other by having the same underground material. The top layer of straw in HMS and BMS was only 5 cm thick.

Interestingly, the measures for BM and BMS were significantly different at V4. In contrast to this, SAW and REC were significantly different at V2 and V3, but at V4 they were not. One possible explanation for that might be derived from the coefficients of determination of the materials. As SAW had R^2^ = 0.7 as opposed to REC with R^2^ = 0.3, the relative increase of cS across the three V-values is greater in SAW than in REC and therefore their absolute stiffness values are closer together at V4. The coefficient of determination was slightly less in BM than in BMS (R^2^ = 0.7 and R^2^ = 0.8) and stiffness values in BM were overall smaller than in BMS. As the increase of stiffness values can be described as linear for both floor types, it is possible that their stiffness values are diverging at rising V-values and therefore differ significantly only at V4. In conclusion, the coefficient of determination as well as the magnitude of stiffness values are considered as characterizing parameters for the relation of two different floors to each other.

Another interesting finding was the small coefficient of determination (R^2^ = 0.1) in SA. Theoretically, the coefficient of determination of an ideal spring would be R^2^ = 0. In this study, SA was, therefore, the material closest to an ideal spring, followed by HM and REC. For practical conditions, this would imply that the height of dropping an object (e.g., the height from which a cow starts the procedure of lying down) is less important in sand compared to floors with a greater coefficient of determination. In general, sand is known as a bedding material with beneficial traits in terms of deformability, which is advantageous, for instance, for preventing hock lesions in cows [11,12]. The results of this study are in line with existing recommendations in literature that sand, offered as a layer of a certain depth, is a favorable lying surface for cows [23].

As previously described, the two softest materials, SAW and SA, required an adapted software version without a correction algorithm. As the threshold for the detection of drops with the default software version was at 30 kN∙m^−1^, the softest material that could be measured with the initial software version was REC. Investigation of differences with ANOVA showed no significant difference between SAW, SA, and REC, except between SAW and REC at V4. Using the updated software version, more caution was necessary in performing measurements to prevent erroneous readings. For instance, movements after arming the device were mistaken as a drop, which would have been automatically excluded with the default setting.

Considering this, we suggest using the initial software version for future studies and practical application and to classify measurements as “below 30 kN∙m^−1^” if the device does not display a stiffness value due to the threshold.

### 4.3. Comparison of the VST with Other Methods

The “knee drop test” [14] is still commonly used by farmers and advisers to compare the softness of different bedding materials. On the one hand, no equipment is needed to conduct this test, but on the other hand, it is a quite subjective estimate. The VST, in contrast, offers objective measures of stiffness and other parameters to characterize the mechanical properties of bedding materials.

Current tools for the assessment of floor properties use static and dynamic measuring methods. One static method described by Tucker et al. [6] is the mixing bowl method, which measures the compressibility (in cm) of bedding materials (wood shavings and straw). A greater compressibility value corresponds with a softer surface.

Considering the measuring method, the CIST is most similar to the VST, as both devices can be referred to as dynamic measuring methods. This tool uses hammers of different weights (e.g., 10 and 20 kg) that are dropped onto the surface within a circumferential groove from a predetermined height of 30 cm. The output is given as the Clegg Impact value (CIV). A large CIV indicates less compressibility. The VST is a modified bowling ball equipped with two accelerometers. As it is operated in free fall, the measurements are not influenced by friction losses.

The direct comparison between the CIV and stiffness values of the VST, however, is not straightforward, as the CIV is a proprietary unit (1 CIV = 10 gravities), and the stiffness of the VST is given as a unit derived from the International System of Units (kN∙m^−1^). Another approach for better comparability of these two devices could use peak acceleration, which is a parameter that is also measured by the VST. In this study, we focused on the parameter “stiffness” to describe the spring rate and thereby the softness of different surfaces.

Using the CIST, Villettaz Robichaud et al. [19] reported significant differences between rubber mats and rubber mats with additional bedding material of different depths (0 cm, 1–2.5 cm, 2.5–5 cm, and >7.5 cm). These results are supported by our findings that rubber mats were significantly harder than the materials that were filled into cubicles.

Other options to assess the quality of the lying surface for cows include animal-mounted sensors, which record lying times of cows. These methods provide individual data on lying behavior, which can be beneficial for more purposes than only assessing the quality of cubicles on the one hand, but require intensive interpretation of data on the other [13].

### 4.4. Practical Considerations and Shortcomings

The VST can be used for a wide range of floor types without changing the equipment. Furthermore, it is easily transportable, as it weighs only 6.15 kg, and has a handle that can be secured for carrying.

Compared to the CIST, measurements with the VST demand more space. Hence, the animals must be moved, which is labor-intensive and can cause stress for the animals [19]. Furthermore, measurements on concrete cannot be conducted with the VST, because it would be damaged when dropping onto floors of that type.

Unlike the CIST, the VST has no display for showing results immediately, but data are directly stored on a micro-SD card. If data were required instantly, it would therefore be necessary to bring a laptop to the measurement site.

One of the main reasons for using the measuring tool was to standardize the height and hence, minimize the number of redundant drops. Preliminary tests had shown that, by using this tool, the number of drops per measurement can be limited to the minimum. Under practical conditions, however, the LED lamps should be sufficient for estimating the heights, after the examiner underwent some training and practice.

There is evidence that the amount and shape of sand and sawdust as bedding material has an impact on the lying behavior of dairy cows [7,24]. Regarding the deformation of sand, the type of sand can have an impact on its characteristics regarding the deformability and therefore, the shape. The sand used in this study was angular and had a particle diameter up to 4 mm, which could lead to sedimentation of smaller particles and therefore, uneven distribution of particles due to the loosening procedure. For this reason, the type of sand used in this study might not be the optimal representation for sand that is recommended for cubicles. Sand with ideal characteristics of deformation for cows should probably be round grained and have less variation in particle size than in our study.

In this project, we did not use another technical device as a gold standard, as we aimed to test the VST under various conditions. However, the stiffness values obtained by the VST can be used as a basis for the objective comparison of bedding materials as used in livestock farming. Future studies could perform a direct comparison of the VST and the CIST.

There are additional factors that have to be considered when assessing the suitability of bedding materials, for example, the surface texture, hygienic conditions, moisture absorption, on-farm feasibility, maintenance effort, costs, and availability in different regions. However, we did not take into account these additional factors in this study, and it was not the purpose of this study to give valid recommendations for the practical on-farm use of a specific bedding material. Our study focused on the evaluation of the VST for measuring the mechanical properties of different floors. Further studies are needed to link objective stiffness values obtained by the VST to other parameters such as cow preference or health parameters. In this context, the VST can be a valuable tool for decision making to improve animal health and welfare.

## 5. Conclusions

This is the first study evaluating the VST on different kinds of bedding materials used in cattle farming. The results show that the actual conditions of bedding materials can be objectively assessed using the VST. Considering the high agreement between measurements obtained with two different devices as well as between two examiners, it can be concluded that the results are reliable. This is of relevance for research and practice. Further research is planned on the practical application of the VST in dairy cow barns in relation to animal health and welfare topics. Using this device with other sensor technologies, such as localization or accelerometer-based systems, might provide further insights, in particular on lying behavior and resting cow comfort. Moreover, animal- (e.g., breed, body weight), environment- (e.g., temperature, humidity) and management- (e.g., stocking density) related changes of bedding quality over time can be demonstrated and could provide, potentially together with data on lying behavior, decision support regarding the need and frequency for new or additional bedding material. Future studies could also evaluate the VST for the application to bedding materials and stall surfaces of other livestock species.

## Figures and Tables

**Table 1:** Mean ± standard deviation (SD), minimum, maximum, and coefficient of variation of calculated stiffness values (cS) on soft rubber mat I (SRM I), soft rubber mat II (SRM II), and hard rubber mat (HRM) at each of the predefined impact velocities (V). N = number of measurements.

Floor	V [m∙s^−1^]	N	Mean ± SD [kN∙m^−1^]	Minimum [kN∙m^−1^]	Maximum [kN∙m^−1^]	Coefficient of Variation
SRM I	2	12	405.2 ± 10.3	390.2	422.2	0.03
	3	12	500.1 ± 13.7	479.3	523.6	0.03
	4	12	594.9 ± 19.0	562.9	625.0	0.03
SRM II	2	12	172.2 ± 7.8	157.7	186.7	0.05
	3	12	309.1 ± 11.0	287.2	321.6	0.04
	4	12	446.1 ± 19.8	408.2	469.5	0.04
HRM	2	12	904.9 ± 40.3	822.6	966.3	0.04
	3	12	1525.1 ± 40.0	1453.9	1575.2	0.03
	4	12	2145.3 ± 49.6	2036.4	2217.9	0.02

**Table 2:** Calculated stiffness values (cS) at predefined impact velocities (V2, V3, and V4) and coefficients of determination (R^2^-values) split by floor type. The different bedding materials are sorted in ascending order according to stiffness: sawdust (SAW), sand (SA), recycled manure solids (REC), horse manure (HM), horse manure with chopped straw (HMS), bark mulch (BM), bark mulch with chopped straw (BMS), soft rubber mat II (SRM II), soft rubber mat I (SRM I), and hard rubber mat (HRM). The unit of impact velocity is m∙s^−1^ (V2 = 2 m∙s^−1^, V3 = 3 m∙s^−1^, V4 = 4 m∙s^−1^), stiffness is given as kN∙m^−1^.

	Impact Velocities			
Floor	V2	V3	V4	R^2^
SAW	9.7	12.6	15.4	0.7
SA	19.2	21.6	24.0	0.1
REC	33.5	36.8	40.1	0.4
HM	48.4	57.6	66.7	0.3
HMS	54.1	70.6	87.1	0.8
BM	78.0	106.4	134.7	0.7
BMS	89.8	129.6	169.4	0.8
SRM II	172.3	309.0	445.8	0.9
SRM I	405.1	500.4	595.7	0.9
HRM	905.7	1524.9	2144.1	>0.9

## References

[1] Berckmans D. (2017). General introduction to precision livestock farming. Anim. Front..

[2] Chapa J.M., Maschat K., Iwersen M., Baumgartner J., Drillich M. (2020). Accelerometer systems as tools for health and welfare assessment in cattle and pigs—A review. Behav. Process..

[3] Charlton G., Bouffard V., Gibbons J., Vasseur E., Haley D., Pellerin D., Rushen J. (2015). Can automated measures of lying time help assess lameness and leg lesions on tie-stall dairy farms?. Appl. Anim. Behav. Sci..

[4] Jensen M.B., Pedersen L.J., Munksgaard L. (2005). The effect of reward duration on demand functions for rest in dairy heifers and lying requirements as measured by demand functions. Appl. Anim. Behav. Sci..

[5] Munksgaard L., Jensen M.B., Pedersen L.J., Hansen S.W., Matthews L. (2005). Quantifying behavioural priorities—Effects of time constraints on behaviour of dairy cows, Bos taurus. Appl. Anim. Behav. Sci..

[6] Tucker C.B., Weary D.M., von Keyserlingk M.A.G., Beauchemin K.A. (2009). Cow comfort in tie-stalls: Increased depth of shavings or straw bedding increases lying time. J. Dairy Sci..

[7] Tucker C.B., Weary D.M. (2004). Bedding on Geotextile Mattresses: How Much is Needed to Improve Cow Comfort?. J. Dairy Sci..

[8] Tucker C.B., Weary D.M., Fraser D. (2003). Effects of Three Types of Free-Stall Surfaces on Preferences and Stall Usage by Dairy Cows. J. Dairy Sci..

[9] Vokey F.J., Guard C.L., Erb H.N., Galton D.M. (2001). Effects of Alley and Stall Surfaces on Indices of Claw and Leg Health in Dairy Cattle Housed in a Free-Stall Barn. J. Dairy Sci..

[10] Delamaire E., Guinard-Flament J. (2006). Increasing Milking Intervals Decreases the Mammary Blood Flow and Mammary Uptake of Nutrients in Dairy Cows. J. Dairy Sci..

[11] Fulwider W.K., Grandin T., Garrick D.J., Engle T.E., Lamm W.D., Dalsted N.L., Rollin B.E. (2007). Influence of Free-Stall Base on Tarsal Joint Lesions and Hygiene in Dairy Cows. J. Dairy Sci..

[12] van Gastelen S., Westerlaan B., Houwers D.J., van Eerdenburg F.J.C.M. (2011). A study on cow comfort and risk for lameness and mastitis in relation to different types of bedding materials. J. Dairy Sci..

[13] Leach K.A., Charlton G.L., Green M.J., Lima E., Gibbons J., Black D., Bradley A.J. (2022). Bedding system influences lying behaviour in dairy cows. Vet. Rec..

[14] Nordlund K., Cook N.B. (2003). A Flowchart for evaluating dairy cow freestalls. Bov. Pract..

[15] Wechsler B., Schaub J., Friedli K., Hauser R. (2000). Behaviour and leg injuries in dairy cows kept in cubicle systems with straw bedding or soft lying mats. Appl. Anim. Behav. Sci..

[16] Henriksen J.C., Munksgaard L. (2019). Validation of AfiTagII, a device for automatic measuring of lying behaviour in Holstein and Jersey cows on two different bedding materials. Animal.

[17] Munksgaard L., Weisbjerg M.R., Henriksen J.C.S., Løvendahl P. (2020). Changes to steps, lying, and eating behavior during lactation in Jersey and Holstein cows and the relationship to feed intake, yield, and weight. J. Dairy Sci..

[18] Fulwider W.K., Palmer R.W. (2004). Use of Impact Testing to Predict Softness, Cow Preference, and Hardening Over Time of Stall Bases. J. Dairy Sci..

[19] Villettaz Robichaud M., Pic A., Delgado H., Adam S., Lacroix R., Pellerin D., Vasseur E. (2020). Short communication: Use of the Clegg hammer measure as an indicator of stall-surface compressibility in tie-stall housing and its relationship with stall comfort. J. Dairy Sci..

[20] Auinger B. (2018). Strohpellets als Pferdeeinstreu im Test—Wasserabsorptionskapazität und Mechanische Eigenschaften im Vergleich zu Stroh und Hobelspänen. Master’s Thesis.

[21] Peham C., Schramel J. (2016). Vorrichtung zur Bestimmung der Elastischen Eigenschaften von Oberflächen und Böden und Verfahren zum Betrieb der Vorrichtung.

[22] Aronhime S., Calcagno C., Jajamovich G.H., Dyvorne H.A., Robson P., Dieterich D., Isabel Fiel M., Martel-Laferriere V., Chatterji M., Rusinek H. (2014). DCE-MRI of the liver: Effect of linear and nonlinear conversions on hepatic perfusion quantification and reproducibility. J. Magn. Reson. Imaging.

[23] Eerdenburg F., Ruud L.E. (2021). Design of Free Stalls for Dairy Herds: A Review. Ruminants.

[24] Drissler M., Gaworski M., Tucker C.B., Weary D.M. (2005). Freestall Maintenance: Effects on Lying Behavior of Dairy Cattle. J. Dairy Sci..

